# Low-cost contact microphones for bedside voice assessment: proof of concept

**DOI:** 10.1007/s00405-025-09970-0

**Published:** 2026-01-31

**Authors:** Adrián Castillo-Allendes, Fernanda Figueroa-Martínez, Lady Catherine Cantor-Cutiva, Mark Berardi, Eric J. Hunter

**Affiliations:** 1 Communicative Sciences and Disorders, Michigan State University, East Lansing, MI US; 2 Communication Sciences and Disorders, The University of Iowa, Iowa City, IA US; 3 Departamento de Fonoaudiología, Pontificia Universidad Católica de Chile, Santiago, Chile; 4 Department of Audiology and Speech-Language Pathology, East Tennessee State University, Johnson City, TN US

**Keywords:** Contact microphone, Voice assessment, Cepstral peak prominence, Bedside assessment, Acoustic analysis

## Abstract

**Purpose:**

To evaluate the proof-of-concept feasibility of using low-cost, commercially available contact microphones (CMs) for bedside voice assessment under simulated hospital noise conditions.

**Methods:**

Two low-cost CMs were tested against a reference accelerometer and headset air microphone using two vocally trained adults. Participants performed sustained vowels, pitch glides, and connected speech under four noise conditions: quiet-lab, quiet-hospital, multi-talker babble, and simulated hospital noise. The selected acoustic parameters, commonly used in clinical assessment, include smoothed cepstral peak prominence (CPPS), fundamental frequency (*f*o), shimmer, jitter, harmonics-to-noise ratio (HNR), noise-to-harmonics ratio (NHR), and low-to-high spectral ratio (L/H ratio). Data were analyzed using generalized estimating equations.

**Results:**

CPPS and *f*o demonstrated no significant device effects and remained stable across noise conditions (*p* > 0.05). Breathy voice significantly reduced CPPS (speech: β = − 0.48, *p* ≤ 0.01; vowel: β = − 0.62, *p* ≤ 0.01) and increased jitter and shimmer (β = 0.74 and 0.75, respectively; *p* ≤ 0.01). Device-related variability was most evident in shimmer and NHR, with accelerometer values differing from CMs. Noise conditions minimally influenced primary measures in CMs compared to the headset microphone.

**Conclusion:**

This feasibility study suggests that low-cost CMs may preserve clinically relevant acoustic measures with stability across noisy conditions. Preliminary findings indicate potential advantages over conventional microphones for bedside voice assessment, though validation with clinical populations in real, rather than staged, conditions is needed.

**Supplementary information:**

The online version contains supplementary material available at 10.1007/s00405-025-09970-0.

## Introduction

Voice is essential for patient communication in clinical settings, enabling individuals to express needs, maintain identity, and retain control during hospitalization [1, 2]. Impaired voice significantly impacts medical outcomes and patient dignity, with vocal recovery as a key indicator of overall rehabilitation progress and emotional well-being [3].

Hospital voice assessment relies predominantly on subjective auditory-perceptual impressions [3, 4]. This subjectivity is compounded by a high ambient noise in clinical environments, where background levels frequently exceed 60 dBA in intensive care units and rehabilitation wards [5–8]. Clinicians report lacking objective bedside voice assessment tools, instead relying on subjective auditory evaluation [3].

Traditional airborne microphones are problematic in hospitals due to noise sensitivity, positioning requirements, and sanitation challenges [8–11]. Environmental noise, mobility restrictions, and lack of portable devices represent major barriers to implementing instrumental voice assessments within real-world hospital workflows [3].

Contact microphones (CMs), particularly piezoelectric throat-mounted sensors, offer promising advantages: reduced background noise sensitivity through skin vibration detection, compact design enabling quick placement, and simplified sanitization [12, 13]. Previous research demonstrated effective signal acquisition in noisy environments and supported automated voice analysis [14–16]. However, most studies have been conducted exclusively in controlled laboratory environments with synthetic stimuli and specialized equipment, limiting clinical generalizability [14, 17]. To our knowledge, no prior studies have directly examined low-cost CMs during human phonation under realistic hospital conditions.

Evaluations of biomedical sensing devices, including microphones, accelerometers, and wearable sensors, follow a staged process that begins with technical verification and analytical validation under controlled conditions, followed by simulated-clinical testing and, finally, clinical validation in patient populations. This structure is described in international standards and in recent regulatory and scientific reviews of physiological monitoring devices [18–20]. Early-stage studies, therefore, focus on device behavior in controlled and simulated conditions before progressing to patient-based validation [21].

Within this framework, this proof-of-concept study evaluated whether low-cost CMs can provide consistent, clinically meaningful acoustic information for bedside voice assessment across staged hospital conditions. By addressing clinical constraints with accessible recording tools, the study establishes preliminary evidence for bedside voice monitoring where conventional instrumentation is impractical [3].

## Materials and methods

### Study design and settings

This pilot study employed a within-subject, repeated-measures design to evaluate two low-cost contact microphones (CMs) for bedside voice assessment under simulated clinical conditions. The study focused on sensor performance rather than participant vocal characteristics and was conducted as part of a larger project on contact microphones in clinical practice, approved by the University of Iowa Institutional Review Board (IRB #202409601).

Recordings were conducted in two distinct environments at the University of Iowa: [1] a sound-treated booth (reverberation: RT60 = 0.1 s; ambient level: Leq = 21 dBA) within the Voice Biomechanics and Acoustics Laboratory, and [2] a hospital procedure room in the Department of Otolaryngology–Head and Neck Surgery (reverberation: RT60 = 0.69 s; ambient level: Leq = 38.6 dBA). The hospital setting reflected realistic acoustic challenges during bedside evaluations. In both environments, identical sensor configurations were tested under quiet conditions and with controlled background noise simulating clinical soundscapes. This design enabled comparison of sensor performance across environments while isolating the effects of ambient noise from other environmental factors.

### Participants and equipment

Two vocally trained adults (female, 31 years; male, 39 years) participated to ensure consistent phonatory productions across conditions. This convenience sample was selected for proof-of-concept evaluation of sensor stability rather than clinical generalizability. No clinical or identifying data were collected; recordings were used solely for sensor evaluation.

Two low-cost CMs, previously acoustically characterized by our group ([CM1] *Alomejor* and [CM2] *DrFeify*), were tested against a reference accelerometer (ACC; 10 mV/g, 1–10 kHz bandwidth; Model 352A24, PCB Piezotronics, Depew, New York, United States) and unidirectional headset microphone (ACM; WH20XLR, Shure Incorporated, Niles, Illinois, United States) serving as the air-conduction reference. It was selected because its directional characteristics and frequency response are well-documented, and it allowed us to examine how contact microphones behave under simulated hospital noise conditions relative to an established air-path recording configuration. Contact microphones were selected based on their commercial availability and signal fidelity, as reported in a previous study [21]. All sensors recorded synchronously (44.1 kHz, 24-bit, WAV format) using an audio interface (Scarlett 4i4, Focusrite PLC, High Wycombe, Buckinghamshire, United Kingdom) and digital audio workstation (REAPER, Cockos Inc., Rosendale, NY, USA).

### Sensor pment and setuplace

CMs were affixed bilaterally to thyroid cartilage lateral laminae using surgical tape (Micropore, 3 M Company, Saint Paul, Minnesota, United States). The reference accelerometer was positioned at midline over the cricothyroid space, approximately 3 cm above the sternal notch. The ACM was positioned 4–5 cm from the participant’s mouth. Skin was cleaned with alcohol wipes before placement to ensure reliable coupling.

Recordings in both environments followed an identical sensor configuration protocol. In the laboratory, participants were seated upright. In the hospital room, participants were reclined at 45° on a standard hospital bed to approximate a typical bedside posture.

**Fig. 1:**
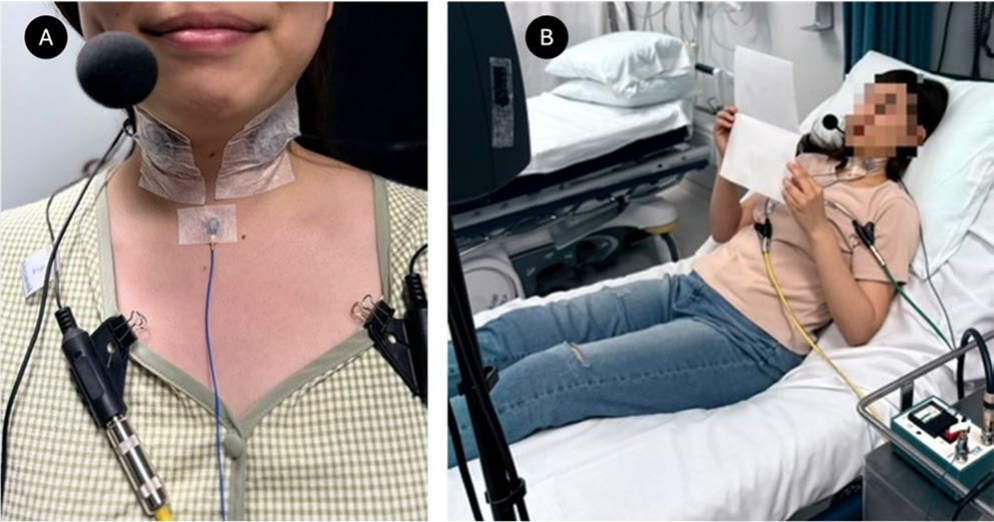
(**A**) Sensor placement including bilateral contact microphones (CM1, CM2), midline accelerometer (ACC), and directional headset microphone (ACM). (**B**) Participant reclined at 45° in the hospital procedure room during recordings

Sensor placement and signal routing were held constant across all tasks and environments, enabling direct comparison of recordings between the sound booth and hospital settings. Sensor placement and participant posture are illustrated in Fig. 1.

### Phonatory tasks and acoustic conditions

Participants completed a structured set of phonatory tasks across two voice qualities: modal and breathy, selected to reflect clinically relevant vocal behaviors. The protocol comprised: (1) sustained vowel phonation (/a/, up to 5 s, 3 repetitions), and (2) connected speech using the first twenty-three syllables of the Rainbow Passage [22, 23].

Tasks were completed in voice-type blocks (modal and breathy), with all repetitions of a given mode recorded consecutively before transitioning to the next. This sequence was preserved across all recording environments.

Three noise conditions were tested: (1) Quiet-lab (~ 21 dBA) or Quiet-hospital (~ 39 dBA), (2) added multi-talker babble (~ 50–55 dBA), and (3) simulated hospital noise (~ 60–65 dBA), which included alarms, equipment, and footfalls. These conditions were tested in two settings: a hospital and a laboratory setting. The selected intensity ranges align with empirical reports of hospital soundscapes, where general wards typically register 50–61 dBA during daytime and ICUs sustain average levels of 52–63 dBA [24–28].

Noise playback (conditions 2 and 3) was delivered through a calibrated loudspeaker (Charge 4, JBL -Harman International Industries, Stamford, Connecticut, United States) positioned 1 m in front of the participant at ~ 1.5 m above floor level. Intensity levels were verified at head position using a Type II sound level meter (Wevosys, Forcheim, Germany) and held constant within each condition.

### Data analysis

Acoustic features were extracted from both vibratory and airborne signals using a custom MATLAB script integrated with Praat (Boersma & Weenink, University of Amsterdam), enabling synchronized multichannel analysis. Parameters commonly used in clinical voice assessment included fundamental frequency of oscillation (*f*o) [29], smoothed cepstral peak prominence (CPPS), low-to-high spectral ratio (LHR), from both sustained vowels and connected speech, with harmonics-to-noise ratio (HNR), noise-to-harmonics ratio (NHR), jitter, and shimmer derived from sustained vowels only.

To examine the effects of device, voice quality, noise condition, and setting, repeated-measures analyses were performed separately for vowels and speech samples. Given non-normal distributions (Shapiro-Wilk test), nonparametric Kruskal-Wallis tests examined device effects within conditions. Associations were further evaluated using Generalized Estimating Equations (GEE) with an exchangeable correlation structure, including variables with *p* < 0.20 at the univariate level. Regression coefficients (β) and standard errors (SE) are reported. Analyses were conducted using SPSS Statistics, Version 24.0 (IBM Corp., Armonk, NY, USA).

## Results

### Acoustic parameters across devices

Analyses of acoustic parameters demonstrated that contact microphones preserved core metrics across noise conditions. Smoothed cepstral peak prominence (CPPS) was significantly lower in breathy phonation for both speech (β = − 0.48, *p* ≤ 0.01) and vowels (β = − 0.62, *p* ≤ 0.01). CPPS standard deviation derived from speech was influenced by voice type (β = − 0.46, *p* ≤ 0.01), while in vowel production, both breathy voice (β = 0.22, *p* ≤ 0.05) and hospital noise (β = 0.22, *p* ≤ 0.05) contributed to increased variability across devices.

Fundamental frequency (*f*₀) extracted from vowels did not show significant associations with device or noise condition. In connected speech, *f*₀ varied primarily with voice type, with higher values observed in breathy phonation (β = 0.22, *p* ≤ 0.01). Standard deviation of *f*₀ from speech did not reach significance, whereas vowel-based *f*₀ standard deviation was significantly higher in breathy productions (β = 0.79, *p* ≤ 0.01).

Other acoustic measures showed significant associations. Shimmer values were lower for the accelerometer compared with *Alomejor* (β = − 0.35, *p* ≤ 0.01), while breathy phonation increased shimmer (β = 0.75, *p* ≤ 0.01). Jitter was significantly higher in breathy phonation (β = 0.74, *p* ≤ 0.01). The accelerometer yielded higher HNR values (β = 0.30, *p* ≤ 0.01), whereas breathy voice reduced HNR (β = − 0.29, *p* ≤ 0.01). NHR increased in breathy phonation (β = 1.34, *p* ≤ 0.01) and under hospital noise (β = 0.54, *p* ≤ 0.01).

The low-to-high ratio (L/H ratio) from speech was influenced by both device and noise condition. Higher values were observed with *DrFeify* (β = 0.10, *p* ≤ 0.01) and babble noise (β = 0.04, *p* ≤ 0.05), while lower values were obtained with the accelerometer (β = − 0.07, *p* ≤ 0.05). Standard deviation of L/H ratio was increased with *DrFeify* (β = 0.25, *p* ≤ 0.01), the accelerometer and headset (β = 0.08, *p* ≤ 0.05), and in hospital recordings compared with laboratory recordings (β = 0.05, *p* ≤ 0.05). In vowel production, significant associations were observed for *DrFeify* (β = 0.27, *p* ≤ 0.01), the headset (β = − 0.39, *p* ≤ 0.01), and hospital setting (β = − 0.48, *p* ≤ 0.01). Detailed results are reported in Table 1.

**Table 1:** Kruskal-Wallis differences of acoustic parameters across devices (accelerometer, *Alomejor*, *DrFeify*, headset) for two voice quality conditions (modal and breathy). Results are presented separately for sustained vowels and connected speech. Values represent means across repetitions.

Parameter	Voice Quality							
	Modal				Breathy			
	Device				Device			
	Accelerometer	Alomejor	DrFeify	Headset	Accelerometer	Alomejor	DrFeify	Headset
*f*o_mean_speech_ (Hz)	173.09	173.77	174.33	173.75	214.08	220.08	214.58	214.61
*f*o_SD_speech_ (Hz)	26.50	26.64	27.13	26.74	30.27	31.21	29.86	29.93
CPPS_mean_speech_ (dB)	8.99	9.49	9.02	9.59	6.01	5.53	5.61	5.77
CPPS_SD_speech_ (dB)**	6.77	6.73	6.94	7.22	4.44	4.02	4.45	4.56
LHR_mean_speech_ (dB) *,**	33.35	36.51	40.24	35.30	33.99	35.50	39.26	32.48
LHR_SD_speech_ (dB) *,**	11.62	11.02	14.46	12.85	12.32	11.05	13.83	10.89
*f*o_mean_vowel_ (Hz)	179.92	179.89	179.79	179.86	198.10	198.20	179.89	198.12
*f*o_SD_vowel_ (Hz)	3.16	2.80	2.18	2.74	4.54	5.15	9.59	4.77
*f*o_kurtosis_vowel_ (Hz)	84.86	63.13	47.32	63.45	17.51	16.55	11.97	15.48
*f*o_skewness_vowel_ (Hz)	6.11	3.85	1.84	3.82	1.97	1.81	0.97	1.70
Jitter (Hz)	0.00	0.00	0.00	0.00	0.01	0.01	0.01	0.01
Shimmer (dB)*,**	0.16	0.22	0.25	0.23	0.29	0.41	0.75	0.46
NHR (dB)**	0.01	0.01	0.01	0.01	0.01	0.03	0.07	0.04
HNR (dB)*,**	32.25	26.61	29.58	25.27	29.08	19.46	18.11	19.68
CPPS_mean_vowel_ (dB)	16.84	16.94	16.74	18.69	9.52	9.09	9.31	9.51
CPPS_SD_vowel_ (dB)	1.29	1.37	1.28	1.42	1.60	1.75	1.55	1.79
LHR_mean_vowel_ (dB) *,**	43.47	45.81	50.43	43.87	41.01	42.78	45.84	39.27
LHR_SD_vowel_ (dB) *,**	6.34	6.53	9.33	4.85	7.55	6.60	7.71	3.99

*f*o = fundamental frequency, *SD* = standard deviation, *CPPS* = cepstral peak prominence smoothed, *LHR* = low-to-high ratio, *HNR* = harmonics-to-noise ratio, *NHR* = noise-to-harmonics ratio, *dB* = decibels, *Hz* = hertz

* Significant differences (*p* < 0.05) for Normal voice quality

** Significant differences (*p* < 0.05) for Breathy voice quality

### Effects on voice quality

When analyses were stratified by phonation type, greater device-related variability was observed for breathy compared to modal voice. CPPS standard deviations from speech and NHR were particularly sensitive to device effects in breathy productions. In addition, shimmer (dB), HNR, and L/H ratio (mean and SD, vowels and speech) consistently showed device-related differences across both voice types. Details are provided in Table 1.

### Effects of noise conditions

Device-related effects were also consistent across noise conditions. Significant differences were observed in L/H ratio (mean and SD, vowels and speech), while CPPS remained interpretable across devices under both babble and hospital noise. Details are summarized in Table 2.

**Table 2:** Kruskal–Wallis differences in acoustic parameters across devices (accelerometer, *Alomejor*,*DrFeify*, headset) under three background noise conditions: no noise, babble noise, and hospital noise. Values are reported for sustained vowels and connected speech

Parameter	Noise Condition											
	Quiet-Hospital Condition				Babble Noise Condition				Hospital Noise Condition			
	Device				Device				Device			
	Accelerometer	Alomejor	DrFeify	Headset	Accelerometer	Alomejor	DrFeify	Headset	Accelerometer	Alomejor	DrFeify	Headset
*f*o_mean_speech_(Hz)	188.11	193.39	188.44	188.33	195.84	196.09	196.44	196.17	196.81	197.95	198.49	198.05
*f*o_SD_speech_ (Hz)	25.89	26.97	25.29	25.57	27.97	27.87	28.43	27.64	31.30	31.41	31.77	31.80
CPPS_mean_speech_ (dB)	7.01	7.58	7.06	7.76	7.85	7.73	7.62	8.04	7.64	7.48	7.27	7.25
CPPA_SD_speech_ (dB)	5.45	5.55	5.49	5.97	5.64	5.38	5.71	5.91	5.72	5.39	5.88	5.79
LHR_mean_speech_ (dB) *,**,***	32.72	35.88	38.49	33.12	34.44	36.61	40.59	33.86	33.85	35.57	40.18	34.70
LHR_SD_speech_ (dB) *,**,***	11.68	11.48	14.32	12.15	11.95	10.78	14.02	12.05	12.27	10.91	14.09	11.41
*f*o_mean_vowel_ (Hz)	180.83	180.80	176.63	180.81	196.96	197.03	186.38	197.00	189.25	189.31	176.51	189.16
*f*o_SD_vowel_ (Hz)	3.66	3.52	7.30	3.56	3.90	4.14	3.18	4.10	4.00	4.26	7.17	3.61
*f*o_kurtosis_vowel_(Hz)	31.10	28.62	13.03	27.30	44.94	41.35	37.30	44.00	77.52	49.54	38.61	47.09
*f*o_skewness_vowel_ (Hz)	2.47	2.24	0.57	2.08	3.72	3.53	2.71	3.70	5.93	2.70	0.94	2.50
Jitter (Hz)	0.00	0.01	0.01	0.01	0.01	0.01	0.01	0.01	0.01	0.01	0.01	0.01
Shimmer (dB)	0.22	0.28	0.53	0.31	0.23	0.30	0.46	0.28	0.22	0.38	0.49	0.46
NHR (dB)	0.01	0.02	0.03	0.02	0.01	0.02	0.03	0.02	0.01	0.03	0.05	0.04
HNR (dB) **	30.60	23.63	23.50	23.51	31.01	23.44	24.42	23.12	30.37	22.03	23.61	20.80
CPPS_mean_vowel_ (dB)	13.47	13.87	13.74	15.11	13.08	12.90	12.92	14.24	12.99	12.29	12.42	12.95
CPPS_SD_vowel_(dB)	1.34	1.40	1.24	1.46	1.48	1.52	1.35	1.46	1.51	1.77	1.64	1.88
LHR_mean_vowel_*,**,***	42.08	43.57	47.13	41.95	42.48	44.52	48.63	40.24	42.16	44.79	48.64	42.52
LHR_SD_vowel_*,**,***	6.46	6.30	7.94	4.74	6.27	6.16	8.21	4.19	8.11	7.23	9.42	4.33

*f*o = fundamental frequency, SD = standard deviation, CPPS = cepstral peak prominence smoothed, *LHR *= low-to-high ratio, *HNR* = harmonics-to-noise ratio, *NHR* = noise-to-harmonics ratio, *dB* = decibels, *Hz* = hertz

* Significant differences (p < 0.05) for Quiet-Hospital condition

** Significant differences (p < 0.05) for Hospital noise condition

*** Significant differences (p < 0.05) for Babble noise condition

### Effects of recording setting

 Table 3 Kruskal–Wallis differences in acoustic parameters across devices (accelerometer, *Alomejor*, *DrFeify*, headset) under two settings: hospital room and laboratory. Values are reported for sustained vowels and connected speech

**Table 3:** Kruskal–Wallis differences in acoustic parameters across devices (accelerometer, *Alomejor*, *DrFeify*, headset) under two settings: hospital room and laboratory. Values are reported for sustained vowels and connected speech

Parameter	Setting							
	Hospital				Laboratory			
	Device				Device			
	Accelerometer	Alomejor	DrFeify	Headset	Accelerometer	Alomejor	DrFeify	Headset
*f*o_mean_speech_ (Hz)	193.09	193.41	194.06	193.86	194.08	198.65	194.86	194.50
*f*o_SD_speech_ (Hz)	27.51	27.53	28.12	27.78	29.26	30.23	28.87	28.90
CPPS_mean_speech_ (dB)	7.51	7.56	7.56	7.48	7.49	7.63	7.07	7.88
CPPS_SD_speech_ (dB)	5.66	5.39	5.79	5.88	5.55	5.48	5.60	5.90
LHR_mean_speech_ (dB) *,**	33.06	38.23	39.99	33.86	34.28	33.62	39.51	33.92
LHR_SD_speech_ (dB) *,**	12.75	11.52	14.21	11.67	11.19	10.50	14.08	12.07
fo_mean_vowel_ (Hz)	190.80	190.82	181.59	190.78	187.22	187.27	178.09	187.20
fo_SD_vowel_ (Hz)	4.08	4.16	5.97	4.11	3.63	3.79	5.80	3.41
fo_kurtosis_vowel_ (Hz)	61.88	55.78	38.24	58.25	40.49	23.90	21.05	20.68
fo_skewness_vowel_ (Hz)	4.89	4.73	2.42	4.72	3.20	0.92	0.40	0.80
Jitter (Hz)	0.01	0.01	0.01	0.01	0.01	0.01	0.01	0.01
Shimmer (dB)*	0.24	0.35	0.51	0.36	0.21	0.29	0.48	0.34
NHR (dB)	0.01	0.02	0.03	0.02	0.01	0.02	0.05	0.03
HNR (dB) **	31.32	23.21	24.51	22.20	30.01	22.85	23.17	22.75
CPPS_mean_vowel_ (dB)	12.79	12.21	12.87	13.54	13.57	13.83	13.18	14.66
CPPS_SD_vowel_ (dB)	1.49	1.53	1.44	1.79	1.39	1.60	1.39	1.41
LHR_mean_vowel_ (dB)*,**	43.19	46.11	49.97	42.36	41.29	42.48	46.30	40.78
LHR_SD_vowel_ (dB) *,**	5.35	4.93	6.55	3.43	8.53	8.20	10.49	5.41

*f*o = fundamental frequency, *SD* = standard deviation, *CPPS* = cepstral peak prominence smoothed, *LHR* = low-to-high ratio, *HNR* = harmonics-to-noise ratio, *NHR* = noise-to-harmonics ratio, *dB* = decibels, *Hz* = hertz

* Significant differences (*p* < 0.05) for Laboratory setting

** Significant differences (*p* < 0.05) for Hospital setting

### Exploratory subject-level analyses

Exploratory analyses conducted by participants indicated that in the female subject, significant device-related differences were observed for jitter (Hz), shimmer (dB), NHR, and HNR (p < 0.05). These effects were not present in the male subject. Device-related differences independent of the participant were consistently observed for the L/H ratio (mean and SD, vowels and speech). These exploratory patterns reflect individual differences rather than gender-specific effects, given the two-participant sample. Details are provided in the Supplementary Information.

## Discussion

 This proof-of-concept study asked a pragmatic question: Can low-cost contact microphones provide stable, clinically interpretable voice metrics under noisy bedside conditions? By comparing two CMs with reference devices across quiet and hospital environments, our preliminary findings suggest that CMs; particulary *DrFeify*, may preserve measurement stability under babble (~50–55 dBA) and simulated hospital noise (~60–65 dBA), whereas the headset microphone behaved as expected for an airborne transducer, admitting ambient interference and inflating variance.

 These findings align with basic transduction physics; skin-coupled sensors are inherently less susceptible to ambient fields than air-conduction microphones [15, 16, 30]. Clinically, this matters because CMs preserve metric interpretability in noisy environments where air microphones fail, such as bedside workflows. This provides proof-of-concept evidence that CMs could be considered as alternatives in hospital conditions where noise control is rarely feasible.

 In our study, CPPS captured the expected modal–breathy contrasts and, when recorded with CMs, remained interpretable under babble and simulated hospital noise relative to the quiet-lab baseline. This is notable because CPPS is one of the most clinically relevant acoustic metrics, strongly correlated with auditory–perceptual judgments of dysphonia severity [31–33]. Previous work has shown that mean CPPS values are context-sensitive, varying with task type (e.g., vowels vs. connected speech), vocal intensity, and background noise [33–36]. Standard deviation values similarly reflect phonatory stability, tending to decrease in steady breathy vowels and increase in more variable or noisy contexts [37]. Clinical studies further show that CPPS provides sensitive and specific diagnostic cutoffs for differentiating disordered from normal voices across languages and speech tasks [38]. Within this framework, our preliminary findings suggest that low-cost CMs can preserve CPPS stability in noisy environments, supporting its use for bedside monitoring of dysphonia severity and treatment response during hospitalization.

 Fundamental frequency provided a complementary signal of robustness. No device effects were observed for vowel-based *f*o, and speech-based *f*o varied primarily with voice type (higher in breathy phonation), not with recording device. Clinically,* f*₀ is the primary acoustic correlate of vocal fold vibration rate and reflects the activity of intrinsic laryngeal muscles such as the cricothyroid and thyroarytenoid [39, 40]. High-pitch *f*₀ probes therefore provide a clinically meaningful way to assess shared neuromuscular mechanisms involved in pitch elevation and airway protection, linking voice assessment directly with swallowing safety [41, 42]. Beyond these applications, our preliminary findings suggest that CMs can capture *f*₀ stably even under noise, enhancing their potential role in bedside monitoring of voice and physiological aspects related to swallowing.

 The present findings apply to measures that primarily reflect glottal-source periodicity and low-frequency spectral energy. Passive skin-coupled contact microphones attenuate higher-frequency components and do not provide reliable information about formant structure or resonance features shaped by the vocal tract [14–16, 21, 43]. Measures that rely on detailed high-frequency spectral information fall outside the scope of what these sensors can reliably represent. However, this feature could be beneficial for sustained voice monitoring in ecological contexts while protecting privacy [44, 45].

 Other acoustic parameters showed device-related differences, particularly those indexing spectral balance (L/H ratio and its dispersion) and spectral noise (HNR/NHR). Our prior characterization of these same CMs revealed attenuated high-frequency responses above 1 kHz, which likely contribute to their impact on tilt- and noise-based measures [46]. These patterns are consistent with prior literature showing that spectral and cepstral measures are sensitive to microphone type, frequency response, and recording conditions [9, 47–50]. and that CMs may color the spectrum and alter tilt/noise parameters relative to air microphones [43]. In our participant-level analysis, L/H ratio effects were observed in both individuals, while variability in jitter, shimmer, NHR, and HNR appeared primarily in the female participant, likely reflecting anatomical or coupling differences rather than sex-specific effects. This highlights the need for standardized placement and attachment protocols and, where spectral balance measures are of interest, lightweight calibration strategies (e.g., brief within-session calibration tokens). Notably, across all devices, *DrFeify*consistently demonstrated better measurement stability, a practical consideration for future device selection in clinical applications.

 Beyond the acoustic findings, this study highlights several practical aspects of clinical feasibility. Low-cost CMs are small and portable, can be connected directly to standard recorders or computers, and require minimal training for clinicians. Their flat surface allows for straightforward cleaning and disinfection between patients, unlike foam-covered headsets or accelerometers that require more complex hygiene protocols. The setup is quick, minimally intrusive, and can be applied during parallel procedures such as endoscopy. Cost considerations are also important: while commercial accelerometers often exceed

 Within the standard development pathway for biomedical sensing devices, early-stage evaluations are conducted under controlled or simulated conditions before progressing to patient-based studies [18–20]. In alignment with this sequence, the present work focused on device behavior during controlled phonation and simulated hospital noise. Because this early-stage protocol did not include patients with dysphonia or postoperative changes, the findings should be interpreted strictly as preliminary feasibility rather than clinical validation. Clinical validation is projected as the next step in this research program.

 Several limitations must be acknowledged. The study included only two vocally trained participants, limiting generalizability to clinical populations where phonation may be more irregular. Additionally, this early-stage design did not include patients with conditions that may alter neck biomechanics or introduce additional sources of low-frequency vibration during phonation, such as postoperative changes, compensatory muscle activity, or irregular vibratory patterns. These factors can affect skin-coupled signals and will need to be examined in dedicated clinical-validation studies with appropriate patient cohorts. Noise was experimentally controlled rather than recorded in real-time clinical context, and placement was standardized, though small differences in coupling pressure and neck anatomy likely influenced certain spectral measures. Chin and cervical angles were also not controlled, and this may contribute to variability in sensor contact and spectral output.

 The 5-second productions were sufficient for computing CPPS and other short-term stability metrics, but this design did not include vocal loading or extended phonation tasks, so our results cannot characterize fatigue- or demand-related changes that have been reported after prolonged or repeated voice use. These aspects will need to be addressed in separate clinical-validation studies using longer or repeated tasks.

 Future studies should expand beyond this proof-of-concept to address three broad priorities. First, larger and more diverse clinical cohorts are needed to establish whether these preliminary patterns hold in populations with dysphonia and in authentic hospital workflows. Second, methodological standardization will be critical to ensure reliable and comparable use across settings. Finally, there is potential to extend the role of contact microphones beyond voice assessment, exploring how their portability and resilience to noise might support broader monitoring of laryngeal and respiratory function, including cough and swallowing. Together, these directions provide a framework for translating proof-of-concept evidence into genuine clinical utility.

### Clinical takeaway

 Preliminary findings suggest that, even in noisy hospitals, low-cost CMs preserve CPPS and *f*o stability. These measures are meaningful and clinically interpretable, offering a potential path toward objective bedside voice assessment and monitoring. For everyday practice, a consistently placed CM focused on CPPS and *f*o offers a pragmatic and scalable path to objective voice assessment.

## Conclusion

 This proof-of-concept study provides preliminary evidence that low-cost contact microphones may offer advantages for voice assessment in noisy clinical environments. In this experiment, CMs preserved the stability of clinically relevant acoustic measures, particularly CPPS and *f*o, across simulated hospital noise conditions better than a conventional headset microphone Initial findings suggest potential for bedside voice monitoring, but further validation is needed with diverse populations and voice disorders in real hospital settings. Understanding device differences will help identify which acoustic parameters contact microphones can reliably capture versus conventional methods. For now, these findings should be seen as preliminary evidence supporting further research rather than immediate clinical use. While contact microphones show promise for accessible voice assessments, more research is necessary to confirm their clinical effectiveness

## Supplementary information

Below is the link to the electronic supplementary material.

ESM 1 (DOCX 33.7 KB)

## Abbreviations

fo: fundamental frequency
SD: standard deviation
CPPS: cepstral peak prominence smoothed
LHR: low-to-high ratio
HNR: harmonics-to-noise ratio
NHR: noise-to-harmonics ratio
dB: decibels
Hz: hertz
