# Supplementary material for: Low-cost contact microphones for bedside voice assessment: proof of concept
Source: Eur Arch Otorhinolaryngol. 2026 Jan 31;283(3):1827–36. doi: 10.1007/s00405-025-09970-0 (PMC13002727; doi:10.1007/s00405-025-09970-0)
Supplement: Supplementary file 1 — (DOCX 33.7 KB) [file 405_2025_9970_MOESM1_ESM.docx]

**Appendix.** Kruskal-Wallis differences in acoustic parameters across devices (ACC, CM1, CM2, Headset) comparing two subjects.

| **Parameter** | **Subjects** | | | | | | | | |
| --- | --- | --- | --- | --- | --- | --- | --- | --- | --- |
|  | **Female** | | | |  | **Male** | | | |
|  | **Device** | | | |  | **Device** | | | |
|  | **Accelerometer** | **Alomejor** | **DrFeify** | **Headset** |  | **Accelerometer** | **Alomejor** | **DrFeify** | **Headset** |
| *f*o_mean_speech_ (Hz) | 245.14 | 246.11 | 244.60 | 245.24 |  | 142.22 | 142.80 | 142.57 | 143.12 |
| *f*o_SD_speech_ (Hz) | 39.40 | 39.67 | 39.82 | 39.45 |  | 17.29 | 17.32 | 16.95 | 17.22 |
| CPPS_mean_speech_ (dB) | 7.24 | 6.94 | 7.50 | 7.66 |  | 7.99 | 7.70 | 7.50 | 7.70 |
| CPPS_SD_speech_ (dB) | 5.02 | 5.16 | 5.22 | 5.53 |  | 5.89 | 6.23 | 5.99 | 6.25 |
| LHR_mean_speech_ (dB) *¥ | 36.75 | 39.30 | 33.95 | 33.46 |  | 35.23 | 40.21 | 33.39 | 34.32 |
| LHR_SD_speech_ (dB) *¥ | 11.94 | 14.97 | 13.53 | 11.81 |  | 10.05 | 13.32 | 10.41 | 11.94 |
| fo_mean_vowel_ (Hz) | 236.79 | 236.65 | 236.72 | 236.69 |  | 141.30 | 123.02 | 141.30 | 141.28 |
| fo_SD_vowel_ (Hz) | 4.04 | 3.32 | 3.53 | 3.52 |  | 3.91 | 8.45 | 4.18 | 3.99 |
| fo_kurtosis_vowel_ (Hz) | 36.96 | 32.66 | 42.03 | 36.93 |  | 42.71 | 26.63 | 60.35 | 42.01 |
| fo_skewness_vowel_ (Hz) | 1.93 | 1.08 | 2.92 | 1.86 |  | 3.73 | 1.74 | 5.16 | 3.67 |
| Jitter (Hz) ¥ | 0.00 | 0.00 | 0.00 | 0.00 |  | 0.01 | 0.01 | 0.01 | 0.01 |
| Shimmer (dB) ¥ | 0.23 | 0.28 | 0.13 | 0.21 |  | 0.41 | 0.71 | 0.32 | 0.48 |
| NHR (dB) ¥ | 0.01 | 0.01 | 0.00 | 0.01 |  | 0.03 | 0.07 | 0.01 | 0.04 |
| HNR (dB) ¥ | 25.59 | 28.13 | 35.15 | 25.68 |  | 20.48 | 19.56 | 26.18 | 19.27 |
| CPPs_mean_vowel_ (dB) | 11.63 | 11.42 | 12.27 | 13.17 |  | 14.41 | 14.64 | 14.10 | 15.03 |
| CPPs_SD_vowel_ (dB) | 1.70 | 1.58 | 1.52 | 1.59 |  | 1.42 | 1.25 | 1.37 | 1.61 |
| LHR_mean_vowel_ (dB) *¥ | 44.21 | 47.83 | 44.36 | 41.50 |  | 44.38 | 48.44 | 40.12 | 41.64 |
| LHR_SD_vowel_ (dB) *¥ | 6.06 | 7.89 | 6.94 | 4.00 |  | 7.07 | 9.15 | 6.95 | 4.84 |

CPPS, Cepstral Peak Prominence Smoothed; dB, Decibels, sound intensity unit; HNR, Harmonics-to-Noise Ratio; Hz, Hertz, unit of frequency cycles per second; LHR, Low-to-High Ratio; NHR, Noise-to-Harmonics Ratio; SD, Standard Deviation.

* Significant differences (p < 0.05) for the male subject.

¥ Significant differences (p < 0.05) for the female subject.
